# Cultivating Creativity and Connection: Goddard House’s Innovative Creative Aging Programs

**DOI:** 10.1093/geroni/igaf122.2134

**Published:** 2025-12-31

**Authors:** Candace Cramer

**Affiliations:** Goddard House, Brookline, Massachusetts, United States

## Abstract

Creative Aging Connections (CAC), a Goddard House initiative, embodies the principles of creative aging, a concept substantiated by Dr. Gene Cohen’s 2006 landmark study which highlights the significant benefits of arts-based learning for the health and wellness of older adults. CAC’s programs leverage these insights to reduce social isolation among under-resourced older adults in Greater Boston, fostering individuality, expression, social connection and belonging through diverse artistic mediums. CAC operates and sponsors creative aging programs that are offered in affordable senior housing buildings and community-based sites in Greater Boston. These programs are informed by our experience engaging with older adults in our assisted living community, and by a literature review and environmental scan of the role and efficacy of art-based interventions in older adult populations, both of which confirm the importance of participant voice and choice, cultural relevance, and high-quality instruction. These elements are crucial in enhancing physical and emotional well-being among older adults. This presentation will show how CAC integrates these research-backed strategies into our creative aging programs, focusing on the positive outcomes of engaging diverse older adults in art forms that resonate with their experiences and needs. One of our sponsored programs, MusicWorks, will present results from a recent qualitative analysis suggesting that music programming offers an outlet for connection as well as positive physical and cognitive outcomes. We will also discuss the challenges and considerations in replicating and scaling such programs, emphasizing the impact of duration, dosage, and participant engagement on the success of these initiatives.

